# Similarities and Differences Between Gerontal and Young Patients with Acute Pancreatitis: Evaluation of Clinical Characteristics and Outcomes

**DOI:** 10.5152/tjg.2022.22227

**Published:** 2022-10-01

**Authors:** Emra Asfuroğlu Kalkan, Çağdaş Kalkan, Sabite Kaçar, Sezgin Barutçu, Mahmut Yüksel, Özge Güçbey Türker, Burak Göre, Tolga Canlı, Umut Asfuroğlu, Berrak Barutçu Asfuroğlu, Mevlüt Hamamcı, Vedat Kılıç, Tankut Köseoğlu, Ersan Özaslan, Bülent Ödemiş, Mesut Kılıç, İlhami Yüksel, Osman Ersoy, Emin Altıparmak, İhsan Ateş, İrfan Soykan

**Affiliations:** 1 Department of Internal Medicine, Ankara City Hospital, Ministry of Health, Ankara, Turkey; 2 Department of Gastroenterology, Ankara City Hospital, Ministry of Health, Ankara, Turkey; 3 Department of Gastroenterology, Gaziantep University Hospital, Gaziantep, Turkey; 4 Department of Radiology, Ministry of Health, Abdulkadir Yüksel Hospital, Gaziantep, Turkey; 5 Department of Gastroenterology, Ankara University Faculty of Medicine, İbni-Sina Hospital, Ankara, Turkey

**Keywords:** Acute pancreatitis, gerontal patients, severe disease

## Abstract

**Background:**

Acute pancreatitis is an abrupt inflammatory disease of the exocrine pancreas and it can occur in different severities. It is becoming more common and more mortal in the gerontal population. The aim of our study was to explore the similarities and differences between young and gerontal patients with acute pancreatitis, with a special emphasis on patients over 80 years of age.

**Methods:**

Medical records of patients (n = 1150) with acute pancreatitis were analyzed retrospectively. Several scoring systems including Bedside index for severity in acute pancreatitis, Ranson’s score, Harmless acute pancreatitis score, Acute Physiology and Chronic Health Evaluation, Balthazar Grade, Glasgow score, and Japanese severity score were applied at admission. Patients were divided into 3 groups; group I, young group (n = 706), if they were aged <65 years; group II, older group (n = 338), if they were aged ≥65 years to <80 years; group III, octogenarian group (n = 106), if they were aged ≥ 0 years.

**Results:**

In total, 1150 patients with acute pancreatitis were analyzed. Octogenarian group (n = 42, 39.6%) showed a more severe acute pancreatitis compared to patients in group I (n = 15, 2.1%) and II (n = 50, 14.8%, *P *< .001). Complications were more common in patients in group III (*P *< .001). Mortality rate was higher in patients in group III (n = 53, 50%) compared to group I (n = 8, 1.1%) and group II (n = 53, 15.7%) (*P *< .001).

**Conclusion:**

Gerontal patients with acute pancreatitis tend to have more severe disease and systemic and local complications. Mortality rates were higher in older patients compared to younger patients.

**Main Points**

- The number of elderly patients with severe acute pancreatitis (AP) was higher than younger patients.
- Gerontal patients with AP tend to have more severe disease, major multiple drug use, and systemic and local complications.
- Disease severity, presence of local/systemic complications, major polypharmacy, and increase in serum creatinine and C-reactive protein levels were risk factors in predicting mortality in octogenarian patients.

## Introduction

Acute pancreatitis (AP) is an abrupt inflammatory disease of the exocrine pancreas causing acinar cell injury with an unpredictable outcome. It is also associated with inflammatory response. Acute pancreatitis can occur in different severities and may present from clinically mild and severe edematous pancreatitis to severe inflammation that can result in death.^1^ Acute pancreatitis is associated with substantial morbidity and mortality causing repeated hospitalizations and impairs long-term quality of life. Acute pancreatitis is seen with increasing frequency and more mortally in the gerontal population.^2-10^ Gullo et al^11^ found that although the mortality rate did not increase, the severity of the disease increased in older patients. In many studies, it has been determined that the age factor is directly related to the need for intensive care unit (ICU) admission and mortality.^10,12-13^ Gerontal patients, especially those over 80 years of age and defined as octogenarians, often present with a severe form of AP and complications such as infected pancreatic necrosis and organ failure that carries a risk of death.^13-16^ In some studies, the situation in local complications was reported different, and no significant difference was found between the gerontal and young populations.^10,17^ Thus, the goals of this research were to explore similarities and differences between gerontal and young patients with AP with a particular emphasis on patients over 80 years of age by means of clinical outcomes. In this study, we studied elderly patients with AP by comparing them with young adult patients in order to determine the diagnostic and prognostic approach to decrease morbidity and mortality.

## Materials and Methods

Medical records of patients (n = 1150) with AP were analyzed retrospectively. Demographic data including age and sex were recorded. Severity and complications of AP, etiology, medications, co-morbidities, biochemical parameters, and computerized tomography findings were obtained from medical records. Other parameters investigated were mortality rate and duration of hospital stay. Several scoring systems including *Bedside index for severity in acute* *pancreatitis (BISAP), Ranson’s score, Harmless acute pancreatitis score (HAPS), Acute Physiology and Chronic Health Evaluation (APACHE II), Balthazar Grade, Glasgow score, *and* Japanese severity score (JSS) *were applied at admission in order to estimate which one best predicts the severity and prognosis of AP. Clinical and demographic data were collected within the first 48 hours after hospital admission. Patients under 18 years of age, patients with active terminal malignancies, pregnant patients, patients with clinical symptoms before 72 hours, patients with metastatic tumors, acquired immunodeficiency syndrome, chronic renal failure, late stage of liver cirrhosis, active tuberculosis, resistant heart failure, immunosuppressive therapy, patients who were diagnosed with chronic pancreatitis, and whose demographic and laboratory parameters could not be reached were excluded from the study. Patients diagnosed as having AP were divided into 3 groups according to their ages at the time of diagnosis. Group I, young group (n = 706), if they were aged <65 years; group II, older group (n = 338), if they were aged ≥65 years to <80 years and group III, octogenarian group (n = 106), if the patients were aged ≥ 80 years or older. Three groups were then compared by means of complications, co-morbidities, medications, in-hospital mortality, length of hospital stay, and by means of the abovementioned scoring systems.

### Definitions

1. **Acute pancreatitis:** Acute pancreatitis is defined according to the Revised Atlanta Criteria of 2012.^18^ In brief, the diagnosis of AP was based on the existence of 2 of the following 3 criteria: 1. acute onset of severe, persistent, epigastric pain often radiating to the back; 2. serum amylase and/or lipase levels at least 3 times the upper normal limit; 3. characteristic findings at the radiological imaging.
2. **Severity:** The severity of AP was classified according to the Revised Atlanta Criteria as mild, moderately severe, and severe. Mild AP; AP without systemic, local complications and organ failure, moderately severe AP; AP was defined as transient organ failure or the presence of local complications or systemic complications; severe AP was defined as permanent organ failure lasting longer than 48 hours.^18^
3. **Medications:** Medications that the patients have already used at the time of examination were expressed under the title of polypharmacy absent (number of drugs <2), minor polypharmacy (number of drugs 2-4), or major polypharmacy (number of drugs ≥5).^19^
4. **Comorbidity:** Heart diseases; chronic lung diseases; acute or chronic liver disease; acute or chronic kidney disease; hematological diseases, including leukemia and lymphoma; diffuse malignancy were defined as chronic comorbid conditions.^20^
5. **Length of hospital stay**: It was calculated from the date of admission to the discharge date.
6. **Scoring systems: **The following scoring systems were calculated in order to define the severity of AP:
  - Bedside index for severity in acute pancreatitis: Includes 5 parameters derived from a retrospective and large-scale study for the early determination of mortality in AP.^21^
  - Ranson’s score: It contains 11 parameters. Five parameters are evaluated at the time of application and 6 parameters are evaluated within the first 48 hours of hospitalization.^22^
  - Harmless acute pancreatitis score: It consists of 3 parameters and is used to identify patients who may not require intensive therapy.^23^
  - Acute Physiology and Chronic Health Evaluation: It consists of 15 parameters evaluated at the time of admission to the hospital.^24^
  - Balthazar Grade: The Balthazar grading scale is a scoring system based on tomographic imaging of the pancreas and used to prognosticate the severity of AP.^25^
  - Glasgow score: It is a scoring system based on 8 parameters looked at 48 hours after hospital admission.^26^
  - Japanese severity score: It consists of 18 parameters.^27^

The present study was established in accordance with Helsinki Declaration, and the ethics committee of the Ministry of Health, Ankara City Hospital has approved the research protocol (number: E1-20-660).

## Statistical Analysis

Analysis of the data was made in Statistical Package for the Social Sciences Statistics version 23 (IBM Corp.; Armonk, NY, USA). A descriptive statistical analysis was performed for baseline characteristics. Descriptive statistics are shown as mean ± standard deviation for variables with normal distribution and as median (min-max) for variables with non-normal distribution. The significance of the difference between the means in the presence of 2 groups was calculated with the *t*-test, and the significance of the difference between the median values was calculated with the Mann–Whitney test. In cases where there are more than 2 groups, the significance of the difference in terms of means was researched with the analysis of variance test, and the significance of the difference in terms of median values was researched with the Kruskal–Wallis test. When there were 2 groups, for variables showing statistical significance, appropriate post hoc tests were used. Receiver operating characteristic (ROC) analysis was used to determine whether BISAP, HAPS, BALTAZAR, RANSON, JSS, GLASGOW, and APACHE score variables were distinctive for the variables of mortality, need for intensive care, systemic disease, and local complications. For the distinctive score values, the cut-off value was determined according to the Youden index (i.e., the value at the highest point of sensitivity and selectivity was accepted as the cut-off). For sensitivity and selectivity, CIs are given for the determined cut-off value. Multivariate logistic regression analysis was performed to determine the independent risk factors affecting mortality in those aged >80 years. In multivariate logistic regression analysis, as a result of univariate analysis, the affecting parameters were determined and taken as candidate variables for multivariate analysis, and the resulting model was obtained by testing with the backward method. The risk coefficients and CIs of the significant variables are indicated. Odds ratios (OR) and CIs for significant parameters were determined. A *P*-value less than .05 was considered significant.

## Results

**Figure 1.**
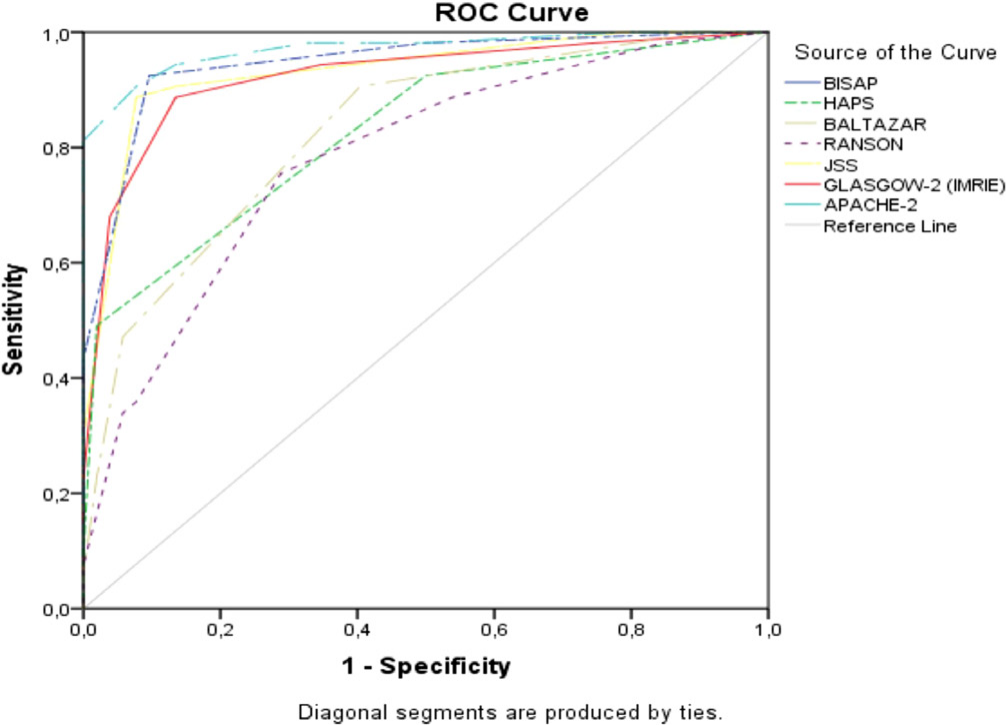
Receiver operating curves (ROC) for scoring systems of acute pancreatitis.

In total, 1150 patients with AP were analyzed in 3 groups (group I: n = 706, <65 years of age, group II: n = 338, 65-80 years, and group III: n = 106, >80 years). Demographic characteristics, disease severity, complications, medication-associated comorbidities, and detailed results of investigated parameters were illustrated in Table 1. The mean age of the patients was 57.85 ± 16.7 years, and 581 (50.5%) were men. As for etiologic causes, biliary causes were found in 89.6% (n = 95) of patients in group III, 72.2% (n=244) in group II, and 47.6% (n = 336) in young patients (*P *< .001). While 101 (14.3%) of patients in group I had hypertriglyceridemia as a cause of AP, 32 (9.5%) patients had alcohol-related AP in group I and 7 (6.6%) of patients in group II were diagnosed as having idiopathic AP. Elderly patients aged >80 years or older (n = 42, 39.6%) showed a more severe AP compared to patients in group I (n = 15, 2.1%) and II (n = 50, 14.8%, *P *< .001). Major polypharmacy was more common in group III (n = 77, 72.6%) compared to patients in groups I and II (*P *< .001). Both systemic and local complications were more common in patients in group III compared to groups I and II (*P *< .001). Mortality rate was higher in patients in group III (n = 53, 50%) compared to group I (n = 8, 1.1%) and group II (n = 53, 15.7%) (*P *< .001). Differences in patients with and without mortality in groups II and III were shown in Tables 2 and 3. There were no differences by means of gender and etiology of AP in group II and age, gender, and etiology of AP in group III. When surviving patients were compared in groups II and III, there were no differences in gender, etiology of AP, systemic and local complications, and ICU admission (Table 4). In multivariate analysis, presence of severe AP (OR: 26.76, 95% CI: 3.16-226.37, *P *< .003), acute necrotic collection and walled-off necrosis (OR: 3.45, 95% CI: 1.01-11.78, *P *< .048), major polypharmacy (OR: 1.18 95% CI: 0.42-3.66, *P *< .043), creatinine >2.4 mg/dL (OR: 2.15, 95% CI: 0.92-20.6, *P *< .031), and C-reactive protein (CRP) >40 (OR: 1.1, 95% CI: 1-2.79, *P *< .001) were found as independent factors affecting mortality in group III (Table 5). All scoring systems used in this study were capable of predicting mortality; however, APACHE predicted mortality with a sensitivity of 90% (95% CI: 80-96) and specificity of 92% (95% CI: 82-97) (Table 6). The ROC analysis of the scoring systems used to determine mortality in the Octogenarian age group is given in Figure 1.

## Discussion

In this study, we carried out a retrospective analysis on gerontal and octogenarian patients with AP by comparing demographic, clinical, and laboratory findings at the onset in 3 different age groups. Patients aged <65 years served as a control group. Moreover, we analyzed factors that might affect mortality and used various risk scoring systems in order to predict mortality. Biliary lithiasis is the most common etiology of AP followed by alcohol-induced AP in all patients (Table 1). However, hypertriglyceridemia was more frequent in younger patients compared to older patients, and idiopathic pancreatitis is in the third place in patients over 80 years (octogenarian) of age. Xin et al investigated 169 patients with severe AP and found that biliary pancreatitis was the most common reason in all patients, but it was more prevalent in the elderly (64.9% vs 37.3%, *P* = .0006).^28^ In the study conducted by Koziel et al^17^ in which 963 patients with a diagnosis of AP were examined, it was determined that cholelithiasis is one of the most important etiological factors of AP among elderly patients. This has been attributed to physiopathological conditions that increase with age, such as lithogenic bile, delayed gallbladder emptying, and dilation of the bile duct.^17^ Classification of the severity of AP is important in terms of providing optimal treatment, identifying patients who will be hospitalized in ICUs, and predicting local and systemic complications that may occur. Elderly patients with AP are at higher risk of serious complications such as pancreatic necrosis and related deaths.^29^ In the current study, the number of elderly patients with severe AP was higher than younger patients. Losurdo et al investigated 42 gerontal patients (65-102 years) and found that elderly patients displayed more severe Atlanta scores and concluded that elderly patients had a more severe course of AP but there were no differences in mortality or number of local complications.^30^ Medications may be one of the etiologic factors of AP in approximately 10% due to polypharmacy.^31^ In our study, older patients were using more medications than the controls. Polypharmacy was observed in 72.6% of patients aged >80 years and 22.2% of patients aged between 65 and 80 years. However, number of patients with polypharmacy was lower in younger patients (n = 198, 17.2%, *P *< .001). It has been reported that local complications such as abscess, pseudocyst, and walled-of necrosis do not increase in older patients compared to young patients; nevertheless, systemic complications are found to be increased in various studies.^17,28^ Kayar et al^32^ reported that the development of local and/or systemic complications was significantly higher in the elderly group and concluded that age and severity were independent risk factors in the development of systemic complications.^[Bibr b32-tjg-33-10-874]^ In our study, both systemic and local complications were significantly encountered in older patients, especially in patients over the age of 80 years.

Although the mortality rate of severe AP has been reported to be as high as about 20%-25%,^33^ recent data indicated that the global mortality rate of patients with AP is predicted to be at 2%-5%.^34^ In our study, mortality rate was 9.9% (n = 114) in the whole study population. However, older patients had a mortality rate of 50% over the age of 80 and 15.7% >65 years of age. These numbers are significantly higher compared to younger patients. In patients who died over the age of 80, duration of hospitalization, the presence of systemic/local complications, and severity of pancreatitis were different from patients who have survived. However, age, gender, presence or absence of polypharmacy, and etiology of pancreatitis were similar. In multivariate analysis of patients over the age of 80 years, presence of moderate/severe pancreatitis, systemic and local complications, major polypharmacy, serum creatinine level (>2.4 mg/dL), and CRP level (>40 mg/dL) remained significantly associated with mortality. Although age is an important risk factor for multisystem organ failure, data regarding increased mortality from AP in older patients remain controversial.^1,10^ Fan et al^35^ investigated 268 patients with AP and reported a mortality rate of 5.9% in younger patients and 21.3% in patients >70 years of age 80. The heterogeneity in the clinical presentation of AP and identifying severe AP patients are important concerns for clinicians. Therefore, an accurate risk scoring system at the onset of the disease is an important issue to guide disease prognosis and clinician treatment choices. We used 7 scoring systems in order to define which one best predicts the mortality in patients with AP over 80 years of age. Acute Physiology and Chronic Health Evaluation scoring system had a sensitivity of 90% and specificity of 92% with a cut-off value of 5.5 in predicting mortality. Teng et al retrospectively investigated 653 patients with AP by means of APACHE II score, Ranson’s, BISAP, HAPS, and SOFA scoring systems and reported that Ranson’s score and APACHE-II showed the highest sensitivity in predicting SAP (92.6% and 80.2% respectively), ICU admission (100%), and mortality (100%). SOFA and BISAP showed lowest sensitivity in predicting SAP (13.6%, 24.7% respectively), ICU admission (40.0%, 25.0% respectively) and mortality (50.0%, 25.5% respectively).^36^

The limitation of this study includes its retrospective nature, and selection bias is always an important issue in studies with a retrospective design. The use of 7 scoring systems in predicting the mortality rate and the number of patients in each group is large enough, and the investigation of multiple factors that might play a role in the prediction of mortality is the strength of our study.

Patients aged 80 years of age and over constitute a unique group compared to other adult groups due to many factors such as decreased cardiac reserve, multiple drug use, and relatively late diagnosis. Biliary complications are at the forefront in this patient group, as in other patient groups, however, according to the literature, cholecystectomy is significantly less performed in this patient group due to comorbid diseases. Therefore, recurrent pancreatitis attacks can be seen more frequently in these patients. Therefore, invasive procedures such as delayed cholecystectomy and ERCP should be planned carefully, especially in octogenarian patients, and the indications should be evaluated periodically. This seems to be the most important step in preventing possible mortality.^37^ As stated in the multivariant analysis performed in our study, a secondary and another important factor is the management of existing polypharmacy in these patients. Multiple drug use, from parasympathomimetic agents to antiaggregant-anticoagulants or analgesics, delays admission to hospital in octogenarian patients, increases possible oddi sphincter dysfunction, bleeding or cardiac-respiratory-renal organ dysfunctions. This prevents invasive interventions that can be done in the early period, as well as delays hospitalization time and therefore hospital stay, and increases deaths.^38^ For this reason, the approach that should be taken is to strictly question the patient in terms of polypharmacy, especially in the octogenarian age group, at the earliest possible stage of admission to the hospital with AP clinic.

In conclusion, this study highlights the differences and similarities between gerontal and younger patients with AP. Gerontal patients with AP tend to have more severe disease, major multiple drug use, and systemic and local complications. Biliary cholelithiasis was the main etiologic factor in all patients, however, hypertriglyceridemia was found in the second line as an etiologic factor in younger patients. Mortality rates and duration of hospitalization were higher in older patients compared to younger patients. Disease severity, presence of local/systemic complications, major polypharmacy, and increase in serum creatinine and CRP levels were risk factors in predicting mortality in patients over the age of 80 years.

## Figures and Tables

**Table 1.** Demographic, Clinical Characteristics, and Investigated Laboratory Parameters and Results of Scoring Systems in Patients Aged Over 65 Years and 80 Years

	All Patients (n = 1150)	<65 (n = 706) (61.4%)	65-80 (n = 338) (29.4%)	≥80 (n = 106)(9.2%)	*P*
**Age **	57.85 ± 16.76	47.62 ± 12.25	70.51 ± 4.15	85.73 ± 3.75	<.001
**Gender **(F/M)	569 (49.5%)/ 581 (50.5%)	358 (50.7%)/ 348 (49.3%)	166 (49.1%)/ 172 (50.9%)	45 (42.5%)/ 61 (57.5%)	.281
**Polypharmacy**					
Absent	541 (47%)	507 (71.8%)	32 (9.5%)	2 (1.9%)	<.001
Minor	411 (35.8%)	153 (21.7%)	231 (68.3%)	27 (25.5%)	<.001
Major	198 (17.2%)	46 (6.5%)	75 (22.2%)	77 (72.6%)	<.001
**Severity**					
Mild AP	658 (57.2%)	509 (72.1%)	135 (39.9%)	14 (13.2%)	<.001
Moderately severe AP	385 (33.5%)	182 (25.8%)	153 (45.3%)	50 (47.2%)	<.001
Severe AP	107 (9.3%)	15 (2.1%)	50 (14.8%)	42 (39.6%)	<.001
**Etiology**					<.001
Biliary cause	675 (58.7%)	336 (47.6%)	244 (72.2%)	95 (89.6%)	
Alcohol-induced	105 (9.1%)	72 (10.2%)	32 (9.5%)	1 (0.9%)	
Post-ERCP	45 (3.9%)	33 (4.7%)	11 (3.3%)	1 (0.9%)	
Hypertriglyceridemia	105 (9.1%)	101 (14.3%)	4 (1.2%)	0	
Hypercalcemia	7 (0.6%)	2 (0.3%)	4 (1.2%)	1 (0.9%)	
Autoimmune pancreatitis	19 (1.7%)	19 (2.7%)	0	0	
Idiopathic	109 (9.5%)	84 (11.9%)	18 (5.3%)	7 (6.6%)	
Medications	20 (1.7%)	11 (1.6%)	9 (2.7%)	0	
Pancreatic duct injury	20 (1.7%)	14 (2.0%)	6 (1.8%)	0	
Anatomic or physiologic pancreatic anomalies	27 (2.3%)	21 (3%)	5 (1.5%)	1 (0.9%)	
Biliary obstruction (IPMN, Pancreas Ca)	18 (1.6%)	13 (1.8%)	5 (1.5%)	0	
Mortality	114 (9.9%)	8 (1.1%)	53 (15.7%)	53 (50%)	<.001
Duration of Hospitalization	7.75 ± 4.3	5.58 ± 3.44	9.86 ± 4.67	15.51 ± 8.25	<.001
ICU/EICU admission	133 (11.6%)	18 (2.5%)	63 (18.6%)	52 (49.1%)	<.001
**Systemic complications**	115 (10%)	17 (2.4%)	52 (15.4%)	46 (43.4%)	<.001
Pleural effusion	76 (6.6%)	10 (1.4%)	36 (10.6%)	30 (28.3%)	
Vascular complication	11 (0.9%)	2 (0.3%)	5 (1.47%)	4 (3.6%)	
Acute respiratory distress syndrome	31 (2.69%)	4 (0.6%)	15 (4.43%)	12 (11.3%)	
Renal insufficiency	40 (3.4%)	5 (0.7%)	19 (5.6%)	16 (15.1%)	
Coronary artery disease	32 (2.78%)	5 (0.7%)	16 (4.7%)	11 (10.3%)	
Multiorgan failure	29 (2.5%)	4 (0.6%)	15 (4.43%)	10 (9.43%)	
**Local complications**	507 (44.1%)	212 (30%)	163 (48.2%)	78 (73.5%)	<.001
Acute peripancreatic fluid collection	397 (34.5%)	192 (27.2%)	141 (41.7%)	54 (50.9%)	
Pancreatic pseudocyst	89 (7.73%)	36 (5.1%)	36 (10.7%)	17 (16%)	
Acute necrotic collection and walled-off necrosis	92 (8%)	11 (1.6%)	42 (12.4%)	39 (36.8%)	
Comorbidities	549 (47.7%)	165 (23.3%)	286 (84.6%)	98 (92.4%)	<.001
Cardiovascular comorbidities	219 (19%)	65 (9.2%)	109 (32.2%)	45 (42.4%)	
Pulmonary comorbidities	66 (5.7%)	3 (0.4%)	28 (8.3%)	35 (33.1%)	
Renal comorbidities	186 (16.1%)	46 (6.5%)	91 (26.9%)	49 (46.2%)	
Neurological	59 (5.1%)	2 (0.3%)	18 (5.3%)	29 (27.3%)	
Diabetes	201 (17.4%)	84 (11.8%)	68 (20.1%)	49 (46.2%)	
Hypertension	268 (23.3%)	96 (13.6%)	94 (27.8%)	78 (73.6%)	
Fatty liver	229 (19.9%)	125 (17.7%)	73 (21.5%)	31 (29.2%)	
BISAP	1 (0-5)	0 (0-4)	1 (0-5)	3 (2-5)	<.001
HAPS	0 (0-3)	0 (0-3)	1 (0-3)	1 (0-3)	<.001
Ranson	2 (0-8)	2 (0-8)	2 (0-8)	4 (1-8)	<.001
APACHE-II	2 (0-12)	1 (0-12)	4 (0-12)	5 (2-12)	<.001
Balthazar	2 (0-8)	1 (0-8)	4 (1-10)	6 (1-10)	<.001
Glasgow	1 (0-8)	0 (0-4)	2 (0-8)	4 (2-8)	<.001
JSS	0 (0-9)	0 (0-3)	3 (1-9)	4 (1-9)	<.001
WBC (×10^3^ /L)	8.23 ± 4.05	8.55 ± 4.15	7.85 ± 3.89	7.32 ± 3.63	<.001
Creatinine (mg/dL)	1.24 ± 0.78	1.09 ± 0.93	1.57 ± 1.22	1.76 ± 0.99	<.001
Albumin (g/dL)	4.02 ± 1.38	4.09 ± 1.7	3.92 ± 0.6	3.83 ± 0.61	.003
C-reactive protein (mg/L)	45.21 ± 33.52	38.33 ± 29.9	50.62 ± 43.9	73.94 ± 60.83	<.001

**Table 2.** Differences Between Patients aged 65-80 Years with and without Mortality

	Mortality (n = 53)	No Mortality (n = 285)	*P*
Age	74.17 ± 3.64	69.83 ± 3.38	<.001
Gender (F/M)	26/27 (49.1%/50.9%)	140/145 (49.1%/50.9%)	.754
Polypharmacy			
Absent	2 (3.8%)	30 (10.5%)	<.001
Minor	29 (54.7%)	202 (70.9%)	<.001
Major	22 (41.5%)	53 (18.6%)	<.001
Acute pancreatitis according to morphological features			
Edematous	10 (18.9%)	194 (68.1%)	<.001
Necrotizing	43 (81.1%)	91 (31.9%)	<.001
According to the severity, acute pancreatitis			
Mild	1 (1.9%)	134 (47%)	<.001
Moderately	12 (22.6%)	141 (49.5%)	<.001
Severe	40 (75.5%)	10 (3.5%)	<.001
Etiology			
Biliary cause	42 (79.2%)	202 (70.9%)	.684
Alcohol-induced	3 (5.7%)	29 (10.1%)	
Post-ERCP	1 (1.9%)	10 (3.5%)	
Hypertriglyceridemia	1 (1.9%)	3 (1.05%)	
Hypercalcemia	1 (1.9%)	3 (1.05%)	
Autoimmune pancreatitis	0	0	
Idiopathic	4 (7.5%)	14 (4.9%)	
Medications	0	9 (3.1%)	
Pancreatic duct injury	0	6 (2.1%)	
Anatomic or physiologic pancreatic anomalies	1 (1.9%)	4 (1.4%)	
Biliary obstruction (IPMN, Pancreas Ca)	0	5 (1.75%)	
Duration of Hospitalization	24.26 ± 14.54	7.19 ± 5.16	<.001
ICU/EICU admission	52 (98.1%)	11 (3.85%)	<.001
Recurrent acute pancreatitis (+/−)	4/49 (7.5%/92.5%)	10/275 (3.5%/96.5%)	.721
Systemic complications	40 (75.4%)	12 (3.1%)	<.001
Pleural effusion	26 (49%)	10 (3.5%)	
Vascular complication	2 (3.8%)	3 (1.05%)	
Acute respiratory distress syndrome	7 (13.2%)	8 (2.8%)	
Renal insufficiency	9 (16.9%)	10 (3.5%)	
Coronary artery disease	8 (15.1%)	8 (2.8%)	
Multiorgan failure	7 (13.2%)	8 (2.8%)	
Local complications	43 (81.1%)	120 (42.1%)	<.001
Acute peripancreatic fluid collection	20 (37.7%)	121 (42.4%)	
Pancreatic pseudocyst	13 (24.5%)	23 (8.1%)	
Acute necrotic collection and walled-off necrosis	17 (32%)	19 (6.6%)	
BISAP	4 (1-5)	2 (1-4)	<.001
HAPS	2 (0-3)	0 (0-3)	<.001
Ranson	4 (1-8)	2 (2-8)	<.001
APACHE-II	6 (2-12)	3 (2-6)	<.001
Balthazar	4 (1-10)	3 (1-7)	<.001
Glasgow	4 (2-8)	2 (2-6)	<.001
JSS	4 (2-9)	3 (2-9)	<.001

**Table 3.** Differences Between Patients Aged 80 Years and Older with and Without Mortality

	Mortality (n = 52)	No Mortality (n = 54)	*P*
Age	85.98 ± 3.95	85.48 ± 3.56	.58
Gender (F/M)	21/31 (40.4%/59.6%)	24/30 (44.4%/55.6%)	.284
Polypharmacy			
Absent	1 (1.9%)	1 (1.9%)	.55
Minor	10 (19.2%)	17 (31.5%)	.414
Major	41 (78.9%)	36 (66.6%)	.082
Acute pancreatitis according to morphological features			
Edematous	15 (28.8%)	25 (46.3%)	.137
Necrotizing	37 (71.1%)	29 (53.7%)	.07
According to the severity, acute pancreatitis			
Mild	1 (1.9%)	13 (24.1%)	<.001
Moderately	13 (25%)	37 (68.5%)	<.001
Severe	38 (73.1%)	4 (7.4%)	<.001
Etiology			
Biliary cause	48 (92.3%)	47 (87%)	.91
Alcohol-induced	0	1 (1.8%)	
Post-ERCP	0	1 (1.8%)	
Hypertriglyceridemia	0	0	
Hypercalcemia	0	1 (1.8%)	
Autoimmune pancreatitis	0	0	
Idiopathic	4 (7.7%)	3 (5.5%)	
Medications	0	0	
Pancreatic duct injury	0	0	
Anatomic or physiologic pancreatic anomalies	0	1 (1.8%)	
Biliary obstruction (IPMN,Pancreas Ca)	0	0	
Duration of Hospitalization	20.06 ± 13.97	10.86 ± 7.98	<.001
ICU/EICU admission	49 (94.2%)	3 (5.5%)	<.001
Recurrent acute pancreatitis (+/-)	3/49 (5.8%/94.2%)	3/51 (5.6%/94.4%)	.45
Systemic complications	32 (61.5%)	14 (25.9%)	<.001
Pleural effusion	21 (40.3%)	9 (16.6%)	
Vascular complication	2 (3.85%)	2 (3.7%)	
Acute respiratory distress syndrome	6 (11.5%)	6 (11.1%)	
Renal insufficiency	8 (15.3%)	8 (14.8%)	
Coronary artery disease	6 (11.5%)	5 (9.25%)	
Multiorgan failure	6 (11.5%)	4 (7.4%)	
Pleural effusion	21 (40.3%)	9 (16.6%)	
Local complications	42 (80.7%)	36 (66.6%)	<.001
Acute peripancreatic fluid collection	30 (57.7%)	24 (44.4%)	
Pancreatic pseudocyst	11 (21.1%)	6 (11.1%)	
Acute necrotic collection and walled-off necrosis	25 (48.1%)	14 (25.9%)	
BISAP	4 (1-5)	2 (1-5)	<.001
HAPS	2 (1-3)	1 (1-3)	<.001
Ranson	6 (2-8)	4 (2-8)	<.001
APACHE-II	8 (2-12)	4 (2-8)	<.001
Balthazar	6 (1-10)	4 (1-10)	<.001
Glasgow	6 (2-8)	2 (2-6)	<.001
JSS	6 (2-9)	4 (2-9)	<.001

**Table 4.** Differences Between Alive Patients Aged 65-80 Years and Over 80 Years

	No Mortality (n = 285) Group 2	No Mortality (n = 54) Group 3	*P*
Age	69.83 ± 3.38	85.48 ± 3.56	<.001
Gender (F/M)	140/145 (49.1%/50.9%)	24/30 (44.4%/55.6%)	.945
Polypharmacy			
Absent	30 (10.5%)	1 (1.9%)	<.001
Minor	202 (70.9%)	17 (31.5%)	
Major	53 (18.6%)	36 (66.6%)	
Acute pancreatitis according to morphological features			
Edematous	194 (68.1%)	25 (46.3%)	<.001
Necrotizing	91 (31.9%)	29 (53.7%)	
According to the severity, acute pancreatitis			
Mild	134 (47%)	13 (24.1%)	<.001
Moderately	141 (49.5%)	37 (68.5%)	<.001
Severe	10 (3.5%)	4 (7.4%)	.013
Etiology			
Biliary cause	202 (70.9%)	47 (87%)	.75
Alcohol-induced	29 (10.1%)	1 (1.8%)	
Post-ERCP	10 (3.5%)	1 (1.8%)	
Hypertriglyceridemia	3 (1.05%)	0	
Hypercalcemia	3 (1.05%)	1 (1.8%)	
Autoimmune pancreatitis	0	0	
Idiopathic	14 (4.9%)	3 (5.5%)	
Medications	9 (3.1%)	0	
Pancreatic duct injury	6 (2.1%)	0	
Anatomic or physiologic pancreatic anomalies	4 (1.4%)	1 (1.8%)	
Biliary obstruction (IPMN, Pancreas Ca)	5 (1.75%)	0	
Biliary cause			
Duration of Hospitalization	7.19 ± 5.16	10.86 ± 7.98	<.001
ICU/EICU admission	11 (3.85%)	3 (5.5%)	.46
Recurrent acute pancreatitis (+/-)	10/275 (3.5%/96.5%)	3/51 (5.6%/94.4%)	.587
Systemic complications	12 (3.1%)	14 (25.9%)	.431
Pleural effusion	10 (3.5%)	9 (16.6%)	
Vascular complication	3 (1.05%)	2 (3.7%)	
Acute respiratory distress syndrome	8 (2.8%)	6 (11.1%)	
Renal insufficiency	10 (3.5%)	8 (14.8%)	
Coronary artery disease	8 (2.8%)	5 (9.25%)	
Multiorgan failure	8 (2.8%)	4 (7.4%)	
Local complications	120 (42.1%)	36 (66.6%)	.532
Acute peripancreatic fluid collection	121 (42.4%)	24 (44.4%)	.102
Pancreatic pseudocyst	23 (8.1%)	6 (11.1%)	.312
Acute necrotic collection and walled-off necrosis	19 (6.6%)	14 (25.9%)	.244
BISAP	2 (1-4)	2 (1-5)	.003
HAPS	0 (0-3)	1 (1-3)	.174
Ranson	2 (2-8)	4 (2-8)	<.001
APACHE-II	3 (2-6)	4 (2-8)	.036
Balthazar	3 (1-7)	4 (1-10)	.003
Glasgow	2 (2-6)	2 (2-6)	.374
JSS	3 (2-9)	4 (2-9)	.31

**Table 5.** Factors Affecting Mortality in the Group Over 80 Years and Their Uni- and Multivariant Analysis

Factors	Univariate Analysis			Multivariate Analysis		
OR	95% CI	*P*	OR	95% CI	*P*
Gender	1.53	0.70-3.32	.285			
Etiology	1.05	0.84-1.31	.677			
According to the severity, acute pancreatitis						
Mild	0.54	0.24-1.22	.138			
Moderately	4.69	0.56-39.52	.015	1.65	0.19-14.12	.04
Severe	169.7	16.14-1769.59	<.001	26.76	3.16-226.37	.003
Systemic complications	70.23	18.14-271.91	<.001		4.3-92.6	.019
Local complications						
Acute peripancreatic fluid collection	2.09	0.96-4.57	.065			
Pancreatic pseudocyst	1.49	0.52-4.28	.454			
Acute necrotic collection and walled-off necrosis	23.33	7.26-75.01	<.001	3.45	1.01-11.78	.048
Polypharmacy						
Minor	1.14	0.07-18.87	.928			
Major	1.82	0.73-4.52	.015	1.18	0.42-3.66	.043
HB	1.02	0.82-1.26	.853			
WBC	1.06	0.95-1.18	.323			
Creatinine (>2.4 mg/dL)	20.32	7.34-56.50	<.001	2.15	0.92-20.6	.031
Albumin	0.77	0.41-1.47	.437			
C-reactive protein (>40 g/L)	2.02	1.01-3.4	<.001	1.1	1-2.79	<.001
BUN	1.02	0.99-1.04	.066			
T.Bil	1.00	0.88-1.14	.944			
D.Bil	1.01	0.86-1.19	.882			
AST	1.00	0.79-1.18	.008			
ALT	1.00	0.82-1.19	.077			
LDH	0.99	0.91-1.12	.186			

**Table 6.** Comparison of BISAP, HAPS, RANSON, JSS GLASGOW, and APACHE Scorings in Determining Mortality in Group 3 Patients

Group 3 Mortality	Area (95% CI)	*P*	Cut-Off Value	Sensitivity (95% CI)	Specificity (95% CI)
BISAP	0.92 (0.90-0.99)	<.001	2.5	0.92 (0.82-0.97)	0.90 (0.79-0.96)
HAPS	0.83 (0.75-0.90)	<.001	1.5	0.49 (0.36-0.62)	0.98 (0.90-0.99)
BALTAZAR	0.83 (0.75-0.91)	<.001	2.5	0.90 (0.80-0.96)	0.60 (0.46-0.72)
RANSON	0.78 (0.69-0.87)	<.001	3.5	0.75 (0.62-0.85)	0.71 (0.58-0.82)
JSS	0.92 (0.89-0.97)	<.001	3.5	0.84 (0.69-0.89)	0.94 (0.84-0.98)
GLASGOW	0.91 (0.87-0.98)	<.001	2.5	0.89 (0.77-0.95)	0.86 (0.75-0.93)
APACHE	0.94 (0.91-1.00)	<.001	5.5	0.90 (0.80-0.96)	0.92 (0.82-0.97)
